# RBP4 Is Associated With Insulin Resistance in Hyperuricemia-Induced Rats and Patients With Hyperuricemia

**DOI:** 10.3389/fendo.2021.653819

**Published:** 2021-06-10

**Authors:** Chan Liu, Xiao-Rong Zhou, Mu-Yao Ye, Xiang-Qing Xu, Yu-Wei Zhang, Hong Liu, Xian-Zhe Huang

**Affiliations:** ^1^ International Medical Department, The Second Xiangya Hospital, Central South University, Changsha, China; ^2^ Department of Geriatrics, The Second Xiangya Hospital, Central South University, Changsha, China; ^3^ Department of Nephrology, The Second Xiangya Hospital, Central South University, Changsha, China; ^4^ Department of Orthopedics, The Second Xiangya Hospital, Central South University, Changsha, China

**Keywords:** hyperuricemia, insulin resistance, insulin receptor substrate, IRS/PI3K/Akt, retinol binding protein 4

## Abstract

**Objective:**

Hyperuricemia (HUA) is strongly associated with abnormal glucose metabolism and insulin resistance (IR). However, the precise molecular mechanism of HUA-induced IR is still unclear. Retinol binding protein 4 (RBP4) has been shown to induce IR in type 2 diabetes mellitus. This study was designed to clarify the relationship between RBP4 and HUA-induced IR and its potential mechanisms.

**Methods:**

Patients with HUA were collected to detect the levels of plasma RBP4 and clinical biochemical indicators. Rats were fed with 10% high yeast and oteracil potassium (300 mg/kg) *via* intraperitoneal injection once daily for eight weeks, and gavage with adenine (100 mg/kg) once daily from the fifth week to induce the HUA model. Glucose consumption testing was performed to determine the capacity of glucose intake and consumption in 3T3-L1 adipocytes. Real-time polymerase chain reaction (RT-PCR) and western blot were used to detect the mRNA and protein level of RBP4 and insulin receptor substrate-phosphatidylinositol 3-kinase-active protein kinase (IRS/PI3K/Akt) signaling pathway-related proteins.

**Results:**

The levels of plasma RBP4 in both HUA patients and HUA rat models were significantly higher than that in the control groups. The level of plasma RBP4 was positively correlated with plasma uric acid, creatinine, fasting insulin, IR index, total cholesterol and triglyceride levels in patients with HUA. In HUA rats, the level of plasma RBP4 was positively correlated with plasma uric acid, IR index, and triglycerides. HUA rats also exhibited IR. After inhibition of RBP4 expression, the phosphorylation levels of the IRS/PI3K/Akt signaling pathway were increased, and IR was significantly improved.

**Conclusion:**

HUA induced IR both *in vitro* and *in vivo*. RBP4 may be involved in HUA-induced IR by inhibiting IRS/PI3K/Akt phosphorylation. Our findings may provide a new insight for the treatment of IR caused by HUA.

## Introduction

In the last few decades, the prevalence of hyperuricemia (HUA) has been rapidly increased worldwide while the age of onset has decreased (1, 2). Accumulating studies suggest that HUA is strongly associated with abnormal glucose metabolism and insulin resistance (IR), which are closely related to the incidence of gout and various metabolic disorders such as metabolic syndrome, hypertension, coronary heart disease and chronic kidney disease (3–5). However, the molecular mechanism of HUA-induced IR is still unclear. Determining the molecular mechanisms of HUA-induced IR has great significance for the effective prevention and treatment of HUA and IR.

Retinol binding protein 4 (RBP4), belonging to the lipocalin family, is mainly synthesized in hepatocytes and the adipose tissues (6). Previous work has shown that RBP4 is regulated by various factors, such as cAMP (7). RBP4 is well-established for its role in transporting retinol from liver to the target tissues. Recently, a growing body of evidence has indicated that RBP4 induces IR and is closely related to type 2 diabetes mellitus, obesity, metabolic syndrome, and cardiovascular disease (8–12). The levels of plasma RBP4 in patients with impaired glucose tolerance and type 2 diabetes mellitus were significantly higher than that in the control (CON) groups (13). Furthermore, decreased RBP4 levels have been verified to be related to improving insulin sensitivity (14–16). However, there are few reports about the relationship between the level of RBP4 and IR in HUA and the mechanisms involved are ill-defined.

In the present study, we found that the levels of plasma RBP4 were significantly higher in patients with HUA and were positively correlated with plasma uric acid (UA), creatinine (Cr), fasting insulin (FIns), HOMA-IR index (HOMA-IR), total cholesterol (TC) and triglycerides (TG). Moreover, we verified that RBP4 promoted HUA-induced IR through inhibition of IRS/PI3K/Akt phosphorylation, which provides a new insight to understand and treat HUA-induced IR.

## Materials and Methods

### Patient Cohorts

Thirty patients with HUA and thirty healthy controls were collected from the medical center at the Second Xiangya Hospital, Central South University. The main enrollment criteria included: i) aged over 18 years; ii) diagnosed with HUA (level of UA in fasting blood >416.5 μmol/L for men and 357 μmol/L for women); and iii) body mass index (BMI) ranged from 18–40 kg/m^2^. Patients with the following conditions were excluded: i) an inability to walk or obvious abnormalities in understanding; ii) obvious clinical liver disease, diabetes or abnormal glucose metabolism, severe renal insufficiency (estimated glomerular filtration rate (eGFR) <30 ml/min/1.73 m^2^); iii) history of major cardiovascular diseases in the past three months, congestive heart failure (NYHA grade III or IV), malignant tumors diagnosed in the past five years, acute or chronic infections, history of organ transplantation or acquired immune deficiency syndrome; and iv) history of alcohol abuse or drug abuse in the past 12 months.

All subjects were investigated one by one using a questionnaire in which name, gender, age, ethnicity, hospital number (inpatient), history of illness and medication were recorded. Blood pressure was measured by benchtop mercury column sphygmomanometer and BMI was also calculated according to the Chinese Adult Overweight and Obesity Prevention and Control Guide (Trial) Standard. Plasma UA, blood urea nitrogen (BUN), Cr, TG, TC, low-density lipoprotein cholesterol (LDL-C), high-density lipoprotein cholesterol (HDL-C), fasting plasma glucose (FPG) and FIns were detected by an automatic biochemical analyzer. The HOMA-IR index was calculated to assess IR status using the following equation: HOMA-IR = FPG × FIns/22.5. Levels of plasma RBP4 were detected by enzyme-linked immunosorbent assay (ELISA). The clinical parameters of all the participants in the study are presented in **Table 1**.

**Table 1:** The clinical parameters of patients with hyperuricemia and controls.

Variable	CON (n = 30)	HUA (n = 30)
Age	51.80 ± 10.28	55.40 ± 15.32
Sex (M/F)	18/12	25/5
Height	165.63 ± 7.55	165.20 ± 7.53
Weight	62.54 ± 10.63	66.17 ± 9.27
BMI (kg/m^2^)	20.70 ± 2.93	24.21 ± 2.62^*^
Systolic pressure (mmHg)	131.80 ± 16.89	142.97 ± 16.58
Diastolic pressure (mmHg)	75.47 ± 11.65	80.13 ± 12.93
UA (μmol/L)	311.11 ± 85.59	588.37 ± 90.57^**^
Cr (μmol/L)	96.27 ± 21.91	124.75 ± 4.36^**^
TC (mmol/L)	4.21 (2.77–6.10)	4.89 (3.51–6.45)^*^
TG (mmol/L)	1.95 (0.76–6.13)	2.48 (0.89–5.22)^*^
HDL-C (mmol/L)	1.04 ± 0.20	1.02 ± 0.22
LDL-C (mmol/L)	2.88 ± 0.65	3.17 ± 0.64
FIns (mmol/L)	6.86 (1.87–19.37)	11.22 (3.8–26.44)^**^
HOMA-IR	1.52 (0.39–4.99)	2.46 (0.62–6.82)^**^
FPG (mmol/L)	4.78 ± 0.68	4.86 ± 0.65

BMI, body mass index; CON, control groups; Cr, creatinine; FPG, fasting plasma glucose; FIns, fasting insulin; HDL-C, high density lipoprotein cholesterol; HOMA-IR, HOMA insulin resistance index; HUA, hyperuricemia; LDL-C, low-density lipoprotein cholesterol; TC, total cholesterol; TG, triglycerides; UA, uric acid. Data are expressed as means ± SEM; ^*^P < 0.05; ^**^P < 0.01.

### HUA Rat Model and Treatment

Male Sprague–Dawley rats (28-week-old, approximately 180–200 g), provided by the Department of Animal Medicine of Central South University, were used for the study. Rats were randomly divided into two groups after one week of adaptive feeding (1): CON group (n = 10) and (2) HUA group (HUA, n = 10). The HUA group was fed with 10% high yeast feed (yeast dry powder: common rat feed = 1:9) and potassium oxonate was injected intraperitoneally at 300 mg/kg once a day for eight weeks. The CON group was fed with a normal diet and an equal dose of sodium carboxymethylcellulose solution was injected intraperitoneally for eight weeks (17). Blood samples were derived from the iliac vein every two weeks to detect renal functions, blood lipids, fasting insulin levels, and tail vein blood was obtained to test plasma glucose. Intragastric administration was started from the fifth week: the HUA group was given 100 mg/kg adenine solution (adenine dissolved in distilled water) once a day with an intragastric dose was 2.5 ml/kg. The CON group was given the same volume of distilled water. After the end of the eight-week experiment with successful model construction, the animals were sacrificed by cervical dislocation and the adipose tissue samples were taken. All experimental procedures were in accordance with the principles of laboratory animal care and approved by the Animal Ethics Committee of the Second Xiangya Hospital, Central South University.

The serum was separated and tested for RBP4 levels by ELISA (rat RBP4 ELISA Test Kit, Abcam, USA). FPG was tested by the Roche blood glucose meter, and FIns, BUN and Cr were detected by ELISA (rat insulin enzyme-linked immunosorbent assay kit, Abcam, USA; rat BUN ELISA Kit, Shanghai Hengyuan Biotechnology, China; Rat Creatinine ELISA Kit, Shanghai Xitang Biological Technology, China, respectively). UA was tested by colorimetry. High-sensitivity C-reactive protein and Cystatin C were detected by rat high-sensitivity C-reactive protein detection kit (colloidal gold method) and rat Cystatin C Assay Kit, respectively. TC and TG were tested by enzymatic method, and LDL-C, HDL-C were tested, respectively, by homogeneous assay and elimination or PPD method, direct assay. In addition, insulin tolerance test, oral glucose tolerance test and HOMA-IR were also calculated.

### Cell Culture and Transfection

The 3T3-L1 preadipocytes were obtained from ATCC (ATCC^®^ CL-173™) and the mycoplasma-free state of the cells was confirmed by using a mycoplasma detection kit before the experiments were conducted. The 3T3-L1 preadipocytes were cultured with 10% fetal bovine serum (FBS), 10 mg/L insulin, 1 μmol/L dexamethasone, and 0.5 mmol/L 3-isobutyl-1-methylxanthine. After being cultured for 48 hours (h), the supernatant was discarded. Cells were then washed with PBS three times and replaced by differentiation medium B (10% FBS, 10 mg/L insulin were added to high glucose DMEM medium). The cells were changed 48 h later in order to be cultured with high-glucose DMEM medium, which could be used in the next experiment, when approximately 90% of the 3T3-L1 preadipocytes would exhibit an adipocyte phenotype. For transient transfection of RBP4 siRNA, RBP4 siRNA oligos (50 nM) were transfected by Lipofectamine 3000 to the 3T3-L1 adipocytes in six-well plates at a density of 2 × 10^5^ cells per well. Differentiated mature 3T3-L1 adipocytes were grouped as needed for the experiment (1): mature 3T3-L1 adipocytes + PBS (2); mature 3T3-L1 adipocytes + 10 mg/dl UA+ scramble siRNA (3); mature 3T3-L1 adipocytes + RBP4 siRNA; and (4) mature 3T3-L1 adipocytes + 10 mg/dl UA + RBP4 siRNA (BeiEr, Changsha, China). The sequences were as follows: scramble siRNA: 5’AGACCTCTCATAGCA GCTGAT 3’; RBP4 siRNA: 5’CAT CCT AGA CGT TGC TAC 3’.

### Real-Time Polymerase Chain Reaction (RT-PCR)

Total RNA was extracted from the cultured 3T3-L1 cells by using trizol reagent (Invitrogen, 15596-026) and RBP4 cDNA was synthesized according to the manufacturer’s instructions (Thermo Fisher Scientific, USA). RT-PCR was performed with the T100 Real-Time PCR Detection System (Bio-Rad, USA) using the SYBR safe (Life Technologies, USA). The reaction conditions were 95°C for 30 s followed by 40 cycles at 95°C for 5 s, and 60°C for 30 s. RBP4 mRNA expression was normalized to the expression of GAPDH using the standard curve method (2^−△△CT^). The sequences of the primers of RBP4 and GAPDH were shown in **Supplementary Table 1**.

### Western Blot

The expression of proteins was determined by western blot as previously described (18). The concentration of protein was detected using a BCA kit (Beyotime, Shanghai, China). Then, 30 μg of total protein was loaded onto 8% SDS polyacrylamide gels and transferred to the polyvinylidene fluoride membrane. The membrane then was blocked by 5% BSA (Sigma) for 1 h and incubated with primary antibodies at 4°C overnight. The next day, the membrane was washed with PBS for three times and then followed by incubation with HRP-labeled goat anti-rabbit IgG (Abcam, America) secondary antibody at 37°C for 1 h. Signals were detected by using the ECL detection kit (Merck Millipore) and normalized to the expression of GAPDH. The antibodies included Total insulin receptor substrate (IRS)-1 (ab52167, 1:1,000, Abcam), phosphorylated IRS-1 (p-IRS-1) at PY896 (sc-560, 1:500, Santa Cruz), total IRS-2 (ab52606, 1:1,000, Abcam), phosphorylated IRS-2 (p-IRS-2) at S731 (sc-1555-R,1:500, Santa Cruz), total PI3K (ab154598,1:1,000, Abcam), phosphorylated PI3K (p-PI3K) at P85 (ab191606, 1:1,000, Abcam), phosphorylated Akt (p-Akt) at Ser-473 (ab192623, 1:1,000, Abcam), GAPDH (ab9485, 1:1,000, Abcam). The absorbance of each band was quantitated using the Image Pro Plus Software.

### Glucose Uptake in 3T3-L1 Adipocytes

The mature 3T3-L1 adipocytes were inoculated into six-well plates and divided into four groups treated with different culture medium containing different drugs. The cells were cultured for an additional 48 h and the glucose content was then determined by a glucose test kit (ab136956, Abcam, USA). The basic value of the glucose content was based on the glucose content of the blank cells with no treatment, and finally, the glucose consumption of the cells in other groups were calculated.

### Statistical Analysis

Data analysis was performed using statistical software (version SPSS 17.0). All normal distribution digital data were expressed as mean ± standard error of the mean (SEM). Non-normal distribution data were subjected to parameter test after data conversion. The gene expression was analyzed by ANOVA variance method. Levene method was used to test the homogeneity of variance, and the comparison between groups was performed by LSD test or Tamhan’s-T2 (M) calibration test. The correlation analysis was performed using Pearson’s correlation analysis and the quantitative relationship between the indicators was analyzed by linear regression analysis. All the experiments were repeated at least three times and *P <*0.05 indicates statistical significance.

## Results

### Relationship Between RBP4 and Metabolic Clinical Indicators in HUA Patients and HUA Rats

To illustrate the relationship between circulating RBP4 and metabolic indicators in HUA diseases, we detected levels of RBP4 and several key metabolic indicators in patients with HUA and HUA rats. According to the results of a previous observational study in our hospital (19), thirty patients with HUA and thirty healthy controls were recruited. The two groups had no significant differences in age, body weight, height, systolic blood pressure, or diastolic blood pressure. BMI was higher in the HUA group than the CON group (24.21 ± 2.62 *vs* 20.70 ± 2.93, *P <*0.05). Besides, the levels of UA, Cr, TC, TG, FIns, and HOMA-IR in the HUA group were significantly higher than those of the CON group, while there were no significant differences of HDL-C, LDL-C and FPG between the two groups (**Table 1**). The levels of plasma RBP4 were positively correlated with UA, Cr, FIns, HOMA-IR, TC, and TG. However, the levels of plasma RBP4 were not significantly associated with FPG, BMI, HDL-C and LDL-C (**Table 2**). Considering plasma RBP4 as a dependent variable, and age, BMI, UA, Cr, TC, TG, FIns, HOMA-IR, and FPG as independent variables for multiple stepwise regression analysis, the analysis results showed that UA, Cr, FIns, and HOMA-IR were independent factors of RBP4 (**Table 3**).

**Table 2:** Correlation between plasma RBP4 and clinical indicators.

Clinical indicators	RBP4	
	R	P-value
UA (μmol/L)	0.518	0.008^**^
Cr (μmol/L)	0.610	0.000^**^
TC (mmol/L)	0.872	0.031^*^
TG (mmol/L)	0.337	0.018^*^
HDL-C (mmol/L)	0.078	0.680
LDL-C (mmol/L)	-0.164	0.369
FIns (mmol/L)	0.260	0.047^*^
HOMA-IR	0.205	0.028^*^
FPG (mmol/L)	0.284	0.129
BMI	0.210	0.275

BMI, body mass index; Cr, creatinine; FPG, fasting plasma glucose; FIns, fasting insulin; HDL-C, high density lipoprotein cholesterol; HOMA-IR, HOMA insulin resistance index; LDL-C, low-density lipoprotein cholesterol; RBP4, retinol binding protein 4; TC, total cholesterol; TG, triglycerides; UA, uric acid. ^*^P < 0.05; ^**^P < 0.01.

**Table 3:** Analysis for correlated factors of plasma RBP4.

**Clinical indicators**	**RBP4**	
	**R^2^**	**P-value**
UA (μmol/)	0.527	0.002^**^
Cr (μmol/L)	0.350	0.000^**^
HOMA-IR	0.624	0.032^*^
FIns (mmol/L)	0.718	0.046^*^

Cr, creatinine; FIns, fasting insulin; HOMA-IR, HOMA insulin resistance index; RBP4, retinol binding protein 4; UA, uric acid. ^*^P < 0.05; ^**^P < 0.01.

Subsequently, we generated a rat model of HUA and analyzed the correlation between the levels of plasma RBP4 and some key metabolic indicators in HUA rats. The results showed that levels of plasma RBP4 were positively associated with UA, TG, and HOMA-IR in HUA rats (correlation index: r = 0.609, 0.715, and 0.262, respectively; *P <*0.05) (**Table 4**). However, there was no significant correlation between the levels of plasma RBP4 and FIns, TC, or high-sensitivity C-reactive protein in HUA rats (*P >*0.05).

**Table 4:** Correlation between plasma RBP4 and clinical indicators in HUA rats.

**Clinical indicators**	**RBP4**	
	**R**	**P-value**
UA (μmol/L)	0.609	0.047^*^
FIns (mmol/L)	−0.472	0.285
TG (mmol/L)	0.715	0.041^*^
TC (mmol/L)	0.20	0.965
Hs-CRP (mg/L)	−0.129	0.784
HOMA-IR	0.262	0.035^*^

FIns, fasting insulin; HOMA-IR, HOMA insulin resistance index; Hs-CRP, high-sensitivity C-reactive protein; HUA, hyperuricemia; RBP4, retinol binding protein 4; TC, total cholesterol; TG, triglycerides; UA, uric acid. ^*^P < 0.05.

### Change of the Levels of Plasma and Adipose RBP4 in HUA Patients and HUA Rats

**Figure 1:**
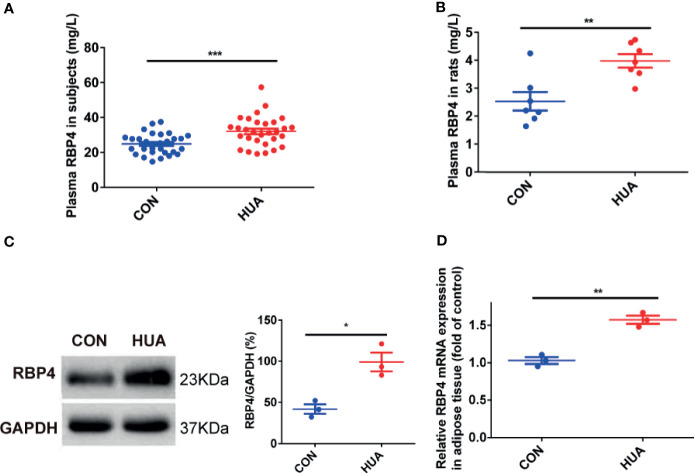
The levels of RBP4 in patients with HUA and HUA rats. **(A)** The levels of plasma RBP4 in thirty patients with hyperuricemia (HUA) and thirty healthy controls (CON). **(B)** Twenty-eight weeks male Sprague–Dawley rats were subjected to high yeast diet and potassium oxonate (300 mg/kg, daily, intraperitoneally) for eight weeks along with adenine (100 mg/kg, by gastrogavage) once daily from the fifth week to induced HUA in rats (HUA, n = 10). Normal diet and vehicle solutions were used in the control group (CON, n = 10). The levels of plasma RBP4 in HUA and CON rats were measured by the specific Elisa kit. **(C)** About 30 μg protein were loaded and the expression of RBP4 in the adipose tissue of rats was determined by western blot (left panel). The representative views are shown in the left panel and densitrometric quantification analysis for RBP4 is shown in the right panel. **(D)** The mRNA expression of RBP4 in the adipose tissue of rats was determined by RT-PCR. Results are represented by mean ± SEM. ^*^ *P* < 0.05, ^**^ *P* < 0.01, ^***^ *P* < 0.001.

Previous works had found that RBP4 is associated with IR in various conditions, such as type 2 diabetes and obesity (9, 20). However, the change of RBP4 expression in HUA is still unclear. Our results showed that the levels of plasma RBP4 in patients with HUA were significantly higher than in the CON groups (32.76 ± 7.59 *vs* 26.19 ± 5.16 mg/L, *P <*0.001) (**Figure 1A**). To further confirm relationship between RBP4 and HUA, we also detected the levels of RBP4 in HUA rats. The results showed that the levels of plasma RBP4 in HUA rats were higher than that in the CON rats (**Figure 1B**). Elevated adipose tissue RBP4 expression has been demonstrated to contribute to IR in type 2 diabetes mellitus and obesity (9, 20). We then detected the expression of RBP4 in the adipose tissue of HUA rats and the results showed that both the mRNA and protein level of RBP4 were markedly increased in adipose tissue of HUA rats compared with those of the CON rats (**Figures 1C, D**). Taken together, these data indicated that the levels of adipose and plasma RBP4 were elevated in both HUA patients and HUA rats.

### Change of Glucose Tolerance and Insulin Sensitivity in HUA Rats

**Figure 2:**
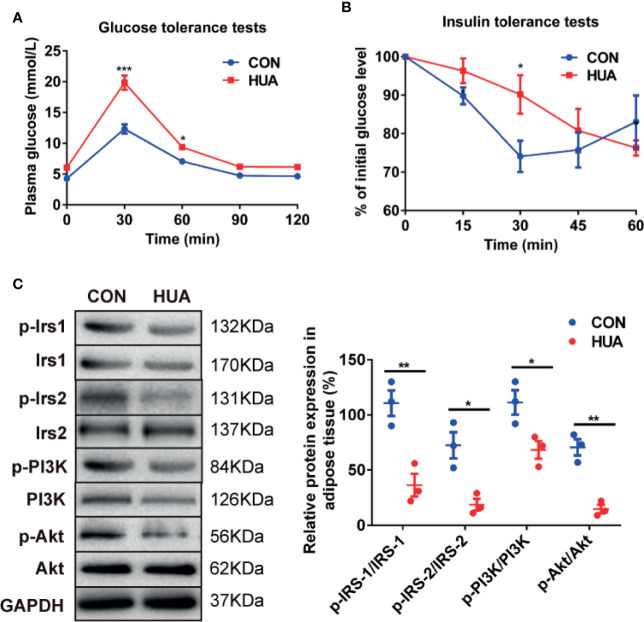
Glucose tolerance and insulin tolerance in HUA rats. **(A)** Glucose tolerance tests were performed to detect glucose tolerance in HUA and CON rats (n = 10 per group). **(B)** Insulin tolerance tests were performed to detect glucose tolerance in HUA and control rats. **(C)** About 30 μg protein were loaded and the expression of p-IRS-1 (PY896), p-IRS-2 (S731), p-PI3K (P85), and p-Akt (Ser473) were measured by Western blot in the adipose tissue of HUA and CON rats. The representative views are shown in the left panel and densitrometric quantification analysis for RBP4 is shown in the right panel. Results are represented by mean ± SEM. ^*^ *P* < 0.05, ^**^ *P* < 0.01, ^***^ *P* < 0.001.

To illustrate the association between HUA and glucose metabolism, we detected the level of glucose tolerance and insulin signaling in HUA rats. The results showed that HUA rats exhibited impaired glucose tolerance when compared with the CON rats, as demonstrated by significantly increased levels of plasma glucose in HUA rats (**Figure 2A**). To analyze insulin sensitivity in rats, we performed insulin tolerance tests. The results showed that HUA rats had a pronounced delay in glucose clearance activity compared to the CON rats, suggesting the existence of IR in HUA rats (**Figure 2B**). In parallel, insulin signaling was examined in HUA rats. Adipose p-IRS-1 (PY896), p-IRS-2 (S731), p-PI3K (P85), and p-Akt (Ser473) expression were strongly repressed in HUA rats when compared to the CON rats, which was accompanied by no significant change of total IRS-1, IRS-2 and PI3K between the two groups (**Figure 2C**). These results demonstrated that HUA rats had impaired glucose tolerance and decreased insulin sensitivity.

### Effects of RBP4 on Glucose Uptake and Insulin Signaling Pathway in 3T3-L1 Adipocytes

**Figure 3:**
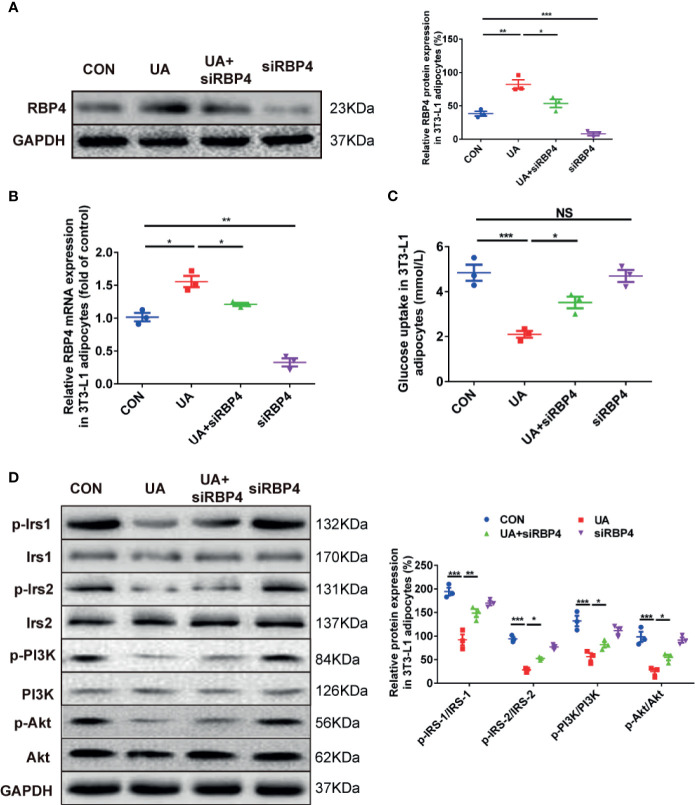
The effects of RBP4 on glucose uptake and phosphorylation of IRS/PI3K/Akt in UA-induced 3T3-L1 adipocytes. 3T3-L1 adipocytes were treated with UA (10 mg/dl), RBP4 siRNA (50nM), or UA+RBP4 siRNA for 48h. **(A)** About 30 μg protein were loaded and the expression of RBP4 was examined by western blot. The representative views are shown in the left panel and densitrometric quantification analysis for RBP4 is shown in the right panel. **(B)** The mRNA expression of RBP4 was determined by RT-PCR. **(C)** Glucose uptake was determined by a glucose test kit. **(D)** About 30 μg protein were loaded and the expression of p-IRS-1 (PY896), p-IRS-2 (S731), p-PI3K (P85), and p-Akt (Ser473) were determined by western blot. The representative views are shown in the left panel and densitrometric quantification analysis for RBP4 is shown in the right panel. Results are represented by mean ± SEM. ^*^ *P* < 0.05, ^**^ *P* < 0.01, ^***^ *P* < 0.001. NS, not significant. All the experiments were repeated three times.

To illustrate whether RBP4 plays a role in regulating glucose tolerance and insulin sensitivity in HUA rats, we evaluated changes of glucose uptake and insulin signaling in UA-induced 3T3-L1 adipocytes in the presence or absence of RBP4 siRNA. The results showed that treatment with UA significantly increased the mRNA and protein expression of RBP4 in 3T3-L1 adipocytes, which was significantly abolished by transfection of RBP4 siRNA (**Figures 3A, B**). Moreover, knockdown of RBP4 blocked UA-induced suppression on glucose uptake in 3T3-L1 adipocytes. However, no difference in glucose uptake was found between the RBP4 siRNA group and the CON group (**Figure 3C**). Mechanically, knockdown of RBP4 reversed UA-induced inhibition of insulin signaling, as demonstrated by increased expression of p-IRS-1 (PY896), p-IRS-2 (S731), p-PI3K (P85), and p-Akt (Ser473) in the UA+RBP4 siRNA group compared to the UA group (**Figure 3D**). These data suggest RBP4 plays a key role in UA suppressed glucose tolerance and insulin sensitivity in 3T3-L1 adipocytes, probably by inhibiting the IRS/PI3K/Akt signaling pathway.

## Discussion

In the present study, we examined the role of RBP4 in HUA-induced IR and its underlying mechanism. The results demonstrate that RBP4 is significantly positively correlated with UA, TG, and HOMA-IR in both HUA rats and patients with HUA. Moreover, the levels of plasma RBP4 are significantly elevated in HUA rats and patients with HUA, which is accompanied by impaired glucose tolerance and reduced insulin sensitivity in HUA rats. Knockdown of RBP4 blocks UA suppressed glucose uptake and reverses UA-induced inhibition on the expression of p-Irs1, p-Irs2, p-PI3K, and p-Akt in 3T3-L1 adipocytes. As a result, our data suggest that RBP4 promotes HUA-induced IR, probably through inhibition of the IRS/PI3K/Akt signaling pathway.

RBP4 has been demonstrated to be associated with impaired glucose hemostasis (20, 21), which is commonly documented in HUA. However, few studies have examined the role of RBP4 in HUA suppressed glucose tolerance and insulin sensitivity. In the present study, the level of plasma RBP4 was demonstrated to be positively associated with glucose metabolic indicators, such as HOMA-IR and Fins, in patients with HUA, suggesting that RBP4 may be involved in the development of HUA-induced IR.

Previous studies had shown that RBP4 was elevated both circulating and in adipose tissue of patients with obesity with or without diabetes (9, 20, 22), which was tightly associated with IR in most, if not all, studies (9, 20, 22, 23). Notably, mice with lower circulating RBP4 exhibited higher insulin sensitivity (24), suggesting that RBP4 is a risk factor for IR. However, circulating and adipose RBP4 levels in HUA are largely unknown. In the present study, we found a pronounced elevation of plasma and adipose RBP4 levels in both HUA rats and patients with HUA. These data suggest that HUA affects the metabolism of RBP4. Interestingly, we found that knockdown of RBP4 significantly blocked UA-induced inhibition of glucose uptake in 3T3-L1 adipocytes, suggesting that RBP4 contributes to UA suppressed glucose tolerance in 3T3-L1 adipocytes. These results are consistent with previous findings that transgenic overexpression of RBP4 induced IR in mice and deletion of RBP4 improved insulin sensitivity (9). Taken together, our data provides possible clues for the potential of RBP4 as targets of insulin resistance in HUA patients. Given the fact the present HUA therapy has obvious effects on lowering urate but little evidence for reducing IR (25), our work provides novel insight in the treatment of HUA by improving IR state.

It has been demonstrated that the IRS/PI3K/Akt signaling pathway plays an important role in the regulation of insulin sensitivity (26). Moreover, adipose RBP4 impairs insulin signaling, which contributes to IR (27). In the present study, we found that UA reduced the phosphorylation of IRS-1, IRS-2, PI3K, and Akt in 3T3-L1 adipocytes, which was blocked by inhibition of RBP4 expression. These results were consistent with the results of Pu et al. documenting that knockdown of RBP4 increased insulin sensitivity by up-regulating the expression of PI3K/Akt insulin signaling pathway-related factors and their phosphorylation levels (28). Our data suggested that RBP4 may contribute to UA-induced inhibition of glucose uptake and IR by inhibiting the IRS/PI3K/Akt signaling pathway. However, more efforts are in needed to illustrate this hypothesis in the future.

The strength of this study was its ability to provide firsthand evidence that RBP4 plays a fundamental role in HUA-induced IR. The study also had some limitations, including the relatively small number of subjects. Another important limitation was that the detailed mechanism was not fully illustrated.

In conclusion, we demonstrated that HUA induced IR both *in vivo* and *in vitro*, accompanied by elevated plasma and adipose RBP4 levels. RBP4 contributes to HUA–induced IR, at least partially by inhibiting the phosphorylation of the IRS/PI3K/Akt signaling pathway in adipocytes, and thereby affecting glucose uptake and inducing metabolic syndrome. Our findings provide a theoretical basis for the treatment of IR in HUA.

## Data Availability Statement

The raw data supporting the conclusions of this article will be made available by the authors, without undue reservation.

## Ethics Statement

The studies involving human participants were reviewed and approved by the Ethics Committee of the Second Xiangya Hospital, Central South University. The patients and participants provided their written informed consent to participate in this study. The animal study was reviewed and approved by the Ethics Committee of the Second Xiangya Hospital, Central South University.

## Author Contributions

X-ZH conceived and designed the experiments. CL performed the experiments, analyzed the data, and prepared all the figures. X-RZ, M-YY, X-QX, Y-WZ, and HL provided technical support. CL wrote the manuscript. All authors contributed to the article and approved the submitted version.

## Funding

This work was supported by the Natural Science Foundation of Hunan Province, China (Grant No. 2018JJ2585) and Health Care Fund of Hunan Province, China (Grant No. B2015-03).

## Conflict of Interest

The authors declare that the research was conducted in the absence of any commercial or financial relationships that could be construed as a potential conflict of interest.

## Abbreviations

HUA, hyperuricemia; CON, control groups; IR, insulin resistance; RBP4, retinol binding protein 4; IRS, insulin receptor substrate; PI3K, phosphatidylinositol 3-kinase; Akt, active protein kinase; UA, uric acid; Cr, creatinine; BUN, blood urea nitrogen; FIns, fasting insulin; HOMA-IR, The HOMA insulin resistance index; TC, total cholesterol; TG, triglycerides; HDL-C, high density lipoprotein cholesterol; LDL-C, low-density lipoprotein cholesterol; BMI, body mass index; FPG, fasting plasma glucose.
