# Supplementary material for: RBP4 Is Associated With Insulin Resistance in Hyperuricemia-Induced Rats and Patients With Hyperuricemia
Source: Front Endocrinol (Lausanne). 2021 Jun 10;12:653819. doi: 10.3389/fendo.2021.653819 (PMC8223863; doi:10.3389/fendo.2021.653819)
Supplement: Supplementary file 1 [file DataSheet_1.pdf]

# Supplementary material

**Supplementary Table 1. The sequence of primers used for qPCR**

| Genes | Sequences |
|---|---|
| RBP4 | Forward: 5’-GGTGAGATGGAGTGGGTGTG-3’ |
| | Reverse: 5’-TGGCGCTCATATGACCCTTC-3’ |
| GAPDH | Forward: 5’-CTCTTCCACCTTCGATGCCG-3’ |
| | Reverse: 5’-GGGGTCTGGGATGGAAATTGT-3’ |
